# HMGB1 released from GSDME-mediated pyroptotic epithelial cells participates in the tumorigenesis of colitis-associated colorectal cancer through the ERK1/2 pathway

**DOI:** 10.1186/s13045-020-00985-0

**Published:** 2020-11-07

**Authors:** Gao Tan, Chongyang Huang, Jiaye Chen, Fachao Zhi

**Affiliations:** Guangdong Provincial Key Laboratory of Gastroenterology, Department of Gastroenterology, Nanfang Hospital, Southern Medical University, Guangzhou, 510515 China

## Abstract

**Background:**

Pyroptosis is a form of proinflammatory gasdermin-mediated programmed cell death. Abnormal mucosal inflammation in the intestine is a critical risk factor for colitis-associated colorectal cancer (CAC). However, it is unknown whether pyroptosis participates in the development of CAC.

**Methods:**

To investigate the role of gasdermin E (GSDME)-mediated pyroptosis in the development of CAC, *Gsdme*^−/−^ mice and their wild-type (WT) littermate controls were challenged with azoxymethane (AOM) and dextran sodium sulfate (DSS) to induce a CAC model. Neutralizing antibodies against high-mobility group box protein 1 (HMGB1) were used to determine the role of HMGB1 in CAC. To identify the role of ERK1/2 in HMGB1-induced colon cancer cell proliferation, we performed western blotting and CCK8 assays using the ERK1/2-specific inhibitor U0126 in CT26 colon cancer cells.

**Results:**

In the CAC model, *Gsdme*^−/−^ mice exhibited reduced weight loss and colon shortening, attenuated rectal prolapse, and reduced tumor numbers and sizes compared to WT littermates. Furthermore, treatment with neutralizing anti-HMGB1 antibodies decreased the numbers and sizes of tumors, ERK1/2 activation and proliferating cell nuclear antigen (PCNA) expression in AOM/DSS-challenged WT mice. In addition, our in vitro experiments demonstrated that HMGB1 induced proliferation and PCNA expression in CT26 colon cancer cells through the ERK1/2 pathway.

**Conclusion:**

GSDME-mediated pyroptosis promotes the development of CAC by releasing HMGB1, which induces tumor cell proliferation and PCNA expression through the ERK1/2 pathway. This finding reveals a previously unrecognized link between pyroptosis and CAC tumorigenesis and offers new insight into CAC pathogenesis.

## Introduction

Colorectal cancer (CRC) is one of the most common types of fatal malignant tumors worldwide [1]. Recently published data show that CRC is the third leading cause of cancer mortality, accounting for 9% of all cancer-related deaths in the USA [2]. More worryingly, the age at onset is becoming younger age. In individuals less than 50 years old, the incidence and death rate have increased by approximately 2% and 1.3% annually in recent years, respectively [3]. As colitis is one of the predisposing risk factors in CRC, CAC accounts for approximately 5% of CRC cases [4].

Prolonged inflammation is one of the characteristics of tumors, and many cancers develop in response to chronic inflammation or display the hallmarks of prolonged inflammation throughout their progression [5–7]. CAC is one of the best examples of tumors that are tightly related to chronic inflammation, which is present in the earliest stage of tumor onset [7]. CAC develops in patients with inflammatory bowel diseases (IBD), including ulcerative colitis (UC) and Crohn’s disease (CD), two clinical phenotypes with risks that are estimated to increase by 0.5–1% per year after 8 to 10 years of IBD [8]. Strikingly, CAC can be delayed or even prevented by treatment with anti-inflammatory drugs, suggesting that inflammatory processes are involved in tumor onset [7].

Pyroptosis was initially considered to be caspase-1-mediated necrosis, mainly in response to bacterial invasion [9]. Recent studies have shown that gasdermin D (GSDMD) and GSDME are cleaved by active caspase-1/4/5/11 and caspase-3, respectively, via the middle linker, releasing their gasdermin-N fragments to induce pyroptosis by perforating the cell membrane [10–12]. This pore-forming activity causes cytoplasmic swelling and releases intracellular contents, such as immunogenic damage-associated molecular patterns (DAMPs) [13, 14]. Therefore, pyroptosis has been redefined as gasdermin-mediated proinflammatory cell death [10, 12]. Because pyroptosis promotes inflammation, it is likely to play an important role in colitis and CAC development. However, it is still not clear whether pyroptosis participates in colitis and CAC development.

DAMPs consist of structurally diverse nonpathogen-derived molecules, and they share some of the following characteristics: (1) they can bind to and activate cell surface or intracellular pattern recognition receptors (PRRs) [15]; (2) they can be not only actively secreted from stressed cells but can also passively released when the plasma membrane is disrupted following certain forms of cell death, such as necrosis, necroptosis, and pyroptosis [16, 17]; and (3) they may switch from a physiological to a proinflammatory function after being released into the extracellular milieu [17]. Various DAMPs have been recognized, including HMGB1, lactoferrin (LTF), S100 proteins A8 and A9 (S100A8/9), IL1a, and IL33 [17]. However, the functional relevance and the effects of these DAMPs on CAC are not entirely clear.

The purpose of this study was to determine the role of gasdermin-mediated pyroptosis in colitis and CAC development. For this purpose, we explored the significance of gasdermin-mediated pyroptosis in experimentally induced colitis and CAC and elucidated its important role in colitis and CAC pathogenesis.

## Methods

### Antibodies and reagents

Anti-GSDME (ab230482) and anti-PCNA antibodies (ab92552) were obtained from Abcam. Anti-ERK (4695), anti-p-ERK (4370), anti-JNK (9252), anti-p-JNK (4668), anti-P38 (8690), and anti-p-P38 (4511) were obtained from Cell Signaling Technology. The neutralizing HMGB1 antibody (ab79823) was purchased from Abcam. Dextran sulfate sodium (DSS) was obtained from MP Biomedicals. AOM (A5486) was purchased from Sigma-Aldrich. Recombinant mouse TNF-α (315-01A) was obtained from PeproTech. Cycloheximide (CHX, C7698), a eukaryote protein synthesis inhibitor, was purchased from Sigma-Aldrich. Recombinant mouse HMGB1 protein (ab181949) was obtained from Abcam. U0126, an ERK1/2 inhibitor, was obtained from InvivoGen. Mouse HMGB1 (E0399m) and IL1a (E0071m) ELISA kits were obtained from EIAab Science Inc, Wuhan. Mouse S100A8 (YXL20093) and S100A9 (YXL20095) ELISA kits were obtained from Yuannuo Science Inc, Chengdu. Mouse LTF (MM-0310M2) and IL33 (MM-0935M2) ELISA kits were obtained from Meimian Science Inc, Guangzhou. Cell counting Kit-8 (CCK8) was obtained from Dojindo Laboratories, Japan.

### Human samples

Endoscopic colonic mucosal biopsy samples were collected from IBD patients and healthy donors at the Nanfang Hospital Gastroenterology Unit. All diagnoses and clinical disease activity assessments were based on a standard combination of clinical, endoscopic and histological assessment and radiologic criteria. All samples were collected from consenting individuals according to the protocols approved by the Ethics Committee of Nanfang Hospital of Southern Medical University. Demographic characteristics are shown in Additional file 1: Table S1.

### DSS-induced colitis in mice

*Gsdme*^*−/−*^ mice (C57BL/6 strain) were kindly provided by Professor Feng Shao (National Institute of Biological Sciences, Beijing, China). *Gsdme*^*−/−*^ mice and WT mice were bred and maintained in a specific pathogen-free facility, and all animal study protocols were approved by the Institutional Animal Care and Use Committee of Southern Medical University. The *Gsdme*^−/−^ and WT mice were littermates and cohoused throughout the experiments. The DSS-induced colitis model was established using a method adapted from a published procedure [18]. Briefly, 8- to 10-week-old male *Gsdme*^−/−^ mice and WT littermate controls were administered 2% DSS dissolved in drinking water. The colon tissues of DSS-challenged mice were embedded in paraffin and stained with H&E. Disease activity index scores and inflammation-associated histopathological assessments were performed according to Nature protocols [18]. Intestinal pathology scores were assessed by two pathologists in a double-blind manner.

### AOM/DSS-induced CAC in mice

CAC was induced with AOM/DSS in mice as described elsewhere [19]. Briefly, 8- to 10-week-old male *Gsdme*^−/−^ mice and WT littermate controls were intraperitoneally injected with AOM (12 mg/kg). Seven days later, the mice were administered 2% DSS dissolved in drinking water for 7 consecutive days, followed by 14 days of regular drinking water for recovery. This same cycle was repeated twice, subsequently followed by regular drinking water until day 84, when these mice were killed. In the antibody-treated groups, neutralizing HMGB1 antibody was intraperitoneally injected at a dose of 200 µg/mouse on days 1, 3, and 5 during DSS treatment. The colons were collected and cut open longitudinally to measure the tumor numbers and sizes.

### Immunohistochemistry (IHC)

IHC was conducted to evaluate the expression levels of GSDME in paraffin-embedded tissues using a specific anti-GSDME antibody. The immunoreactive score (IRS) of each sample was obtained by multiplying the score for the percentage of positive cells (0: no positive cells; 1: < 10% positive cells; 2: 10–50% positive cells; 3: 51–80% positive cells; and 4: > 80% positive cells) and the score for the staining intensity (0: no color reaction; 1: mild reaction; 2: moderate reaction; and 3: intense reaction). All sample IRSs were determined by two independent pathologists who were blinded to both the origin of the samples and the patient outcomes.

### Isolation of intestinal epithelial cells

Biopsy samples were processed immediately, and IECs were purified using enzymatic digestion as previously described [20]. Briefly, colonic tissues from the WT and KO mice were repeatedly washed in HBSS containing 1 mM DTT and 1% penicillin/streptomycin (Sigma-Aldrich). Then, IECs were isolated by incubation in HBSS (Invitrogen/GIBCO) containing 3 mM EDTA (Sigma-Aldrich). After enzymatic digestion, a 40% Percoll Plus solution (GE Healthcare) was used to remove the mononuclear cells, red blood cells, and dead cells.

### Real-time PCR

Quantitative real-time PCR (qRT-PCR) was performed as previously described [21]. The mRNA levels of target genes were normalized to that of GAPDH. The primers used are shown in Additional file 1: Table S2.

### Cell culture

The mouse colon cancer cell line CT26 was obtained from Guangdong Provincial Key Laboratory of Gastroenterology, Southern Medical University. CT26 cells were cultured in Dulbecco’s modified Eagle’s medium (DMEM, Gibco) supplemented with 10% fetal bovine serum (Gibco) in 5% CO_2_ at 37 °C.

### CCK8 assay

Cell growth was assessed using the CCK8 assay. Briefly, CT26 cells (1 × 10^3^ cells/well) were seeded in 96-well plates. The next day, the cells were treated with 1 µg/ml recombinant mouse HMGB1 protein. After 48 h, 10 µl of CCK8 solution was added to each well and incubated for 1 h before the absorbance was measured at 450 nm.

### Western blotting

Western blot analysis was performed as previously described [22]. GAPDH was used as an endogenous control. Anti-GSDME, anti-p-p38, total p38, p-JNK, total JNK, p-ERK1/2, total ERK1/2, and anti-PCNA antibodies were diluted 1:1000. Anti-GAPDH was diluted 1:2000. Secondary antibodies were diluted 1:4000. ImageJ software was used to quantify and analyze the density of the protein bands.

### ELISA

For ELISA analysis, IECs from *Gsdme*^−/−^ mice and WT littermate controls that were treated with DSS for 7 days were isolated using isolation buffer (5 mM EDTA and 1 mM DTT) and then washed with PBS containing penicillin (100 U/ml) and streptomycin (100 mg/ml) 3 times. Finally, the cells were plated in dishes with DMEM containing 10% FBS for 12 h, and the cell medium was used for ELISA analysis. In our in vitro experiment, isolated IECs from *Gsdme*^−/−^ mice and WT littermate controls were treated with TNF-α (50 ng/ml) plus CHX (20 µg/ml) for 12 h, and then, the culture supernatant was collected for ELISA analysis.

### Statistical analysis

Unless otherwise indicated, statistical analyses were performed using GraphPad Prism software. Except as otherwise indicated, all experimental data are presented as the mean ± SEM, and statistical significance was determined using a two-tailed Student’s t test. *P* values < 0.05 were considered significant.

## Results

### GSDME correlates with mucosal inflammation in IBD patients

**Fig. 1:**
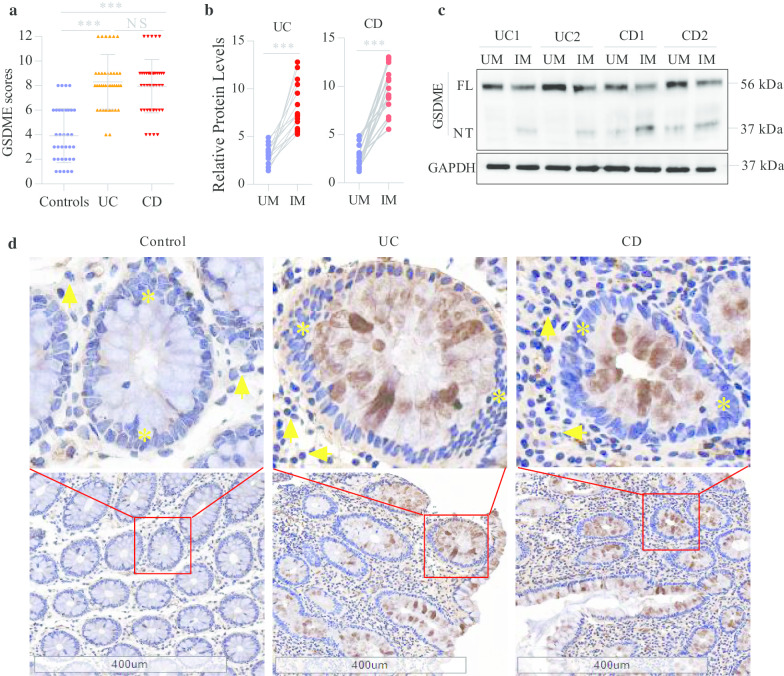
The clinical correlation of GSDME in IBD patients. **a** IHC analyses of GSDME protein in the colonic mucosa from healthy controls (*n* = 40), UC patients (*n* = 42) and CD patients (*n* = 43). **b** *UM* un-inflamed mucosa, *IM* inflamed mucosa. Relative GSDME-NT levels (GSDME-NT/GAPDH) in the inflamed and un-inflamed colonic mucosa from UC patients (*n* = 15) and CD patients (*n* = 15) were further compared as determined by the paired *t* tests. **c** Representative immunoblot images of the colonic mucosa from UC and CD patients. The full length of GSDME (GSDME-FL) is shown in 56 kDa and GSDME-NT in 37 kDa. **d** Representative IHC images of the colonic mucosa from healthy controls, UC and CD patients. Scale bars: 400 μm. Yellow asterisks: epithelial cells. Yellow arrows: lymphocytes. NS, not significant; ***P < 0.001

The abnormal chronic mucosal inflammation in IBD makes it a refractory intestinal disease [23, 24]. Pyroptosis is a form of proinflammatory cell death [14]. However, it is not yet known whether pyroptosis is involved in the development of mucosal inflammation in IBD. To investigate the involvement of pyroptosis in mucosal inflammation in IBD, we measured the expression levels of gasdermins in the mucosa of IBD patients and found that the protein levels of GSDME were significantly increased in the colonic mucosa of IBD patients compared to healthy controls (Fig. 1a, d). Importantly, the pyroptosis-inducing fragment GSDME-NT could be detected in the inflamed colonic mucosa of IBD patients (Fig. 1b, c) but was not detected in the uninflamed mucosa (Fig. 1c). In addition, we found that GSDME was mainly located in the epithelial cells of the mucosa (Fig. 1d), which was consistent with previous findings showing that gasdermins are mainly expressed in the epithelium of the gastrointestinal tract and skin [25, 26]. Taken together, these results suggest that GSDME-mediated epithelial cell pyroptosis correlates with mucosal inflammation in IBD patients.

### GSDME deletion mitigates DSS-induced colitis

**Fig. 2:**
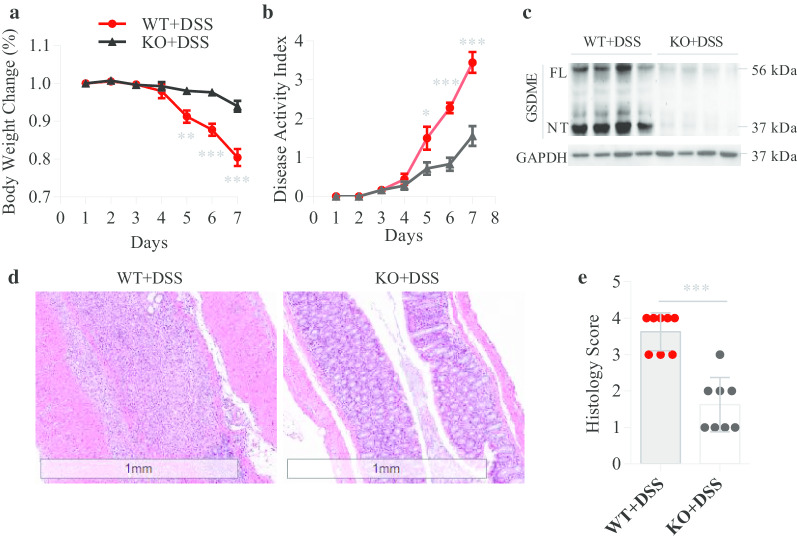
GSDEM deletion mitigates DSS-induced colitis. *Gsdme*^−/−^ (KO) mice and wild-type (WT) littermate controls were treated with 7-day DSS to induce acute colitis (*n* = 8 per group); then, body weight change (**a**) and disease activity index (**b**) were measured daily. **c** Immunoblot analyses of GSDME and GSDME-NT in the colonic mucosa of DSS mice. The full length of GSDME (GSDME-FL) is shown in 56 kDa and GSDME-NT in 37 kDa. **d**, **e** Representative HE-stained sections of middle colon tissues collected at day 7. Histopathology change reflected by semiquantitative scores (Scale bars: 1 mm). All data shown are representative of three independent experiments. *P < 0.05; **P < 0.01; ***P < 0.001

To investigate the role of GSDME in colitis development, we established a DSS-induced colitis model with *Gsdme*^−/−^ mice and WT littermate controls and found that *Gsdme*^−/−^ mice were resistant to DSS-induced colitis, exhibiting less weight loss and disease activity index than WT controls (Fig. 2a, b). Moreover, histological examination revealed that WT mice had severe mucosal inflammation in the colon, while the severity of mucosal inflammation was significantly decreased in *Gsdme*^−/−^ mice (Fig. 2d, e). In addition, the levels of GSDME-NT were notably high in the colonic mucosa of WT mice, but GSDME-NT was not detected in the colonic mucosa of *Gsdme*^−/−^ mice (Fig. 2c). These results indicate that GSDME-mediated pyroptosis plays an important role in the development of colitis.

### GSDME increases HMGB1 release from IECs

**Fig. 3:**
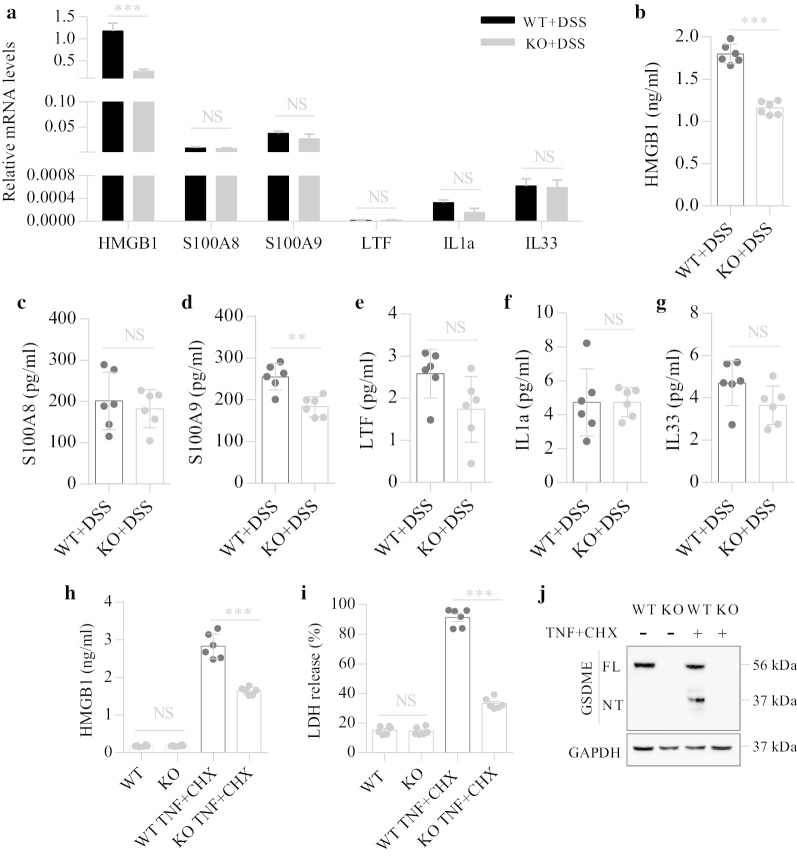
GSDME-mediated IEC pyroptosis increases the release of HMGB1. **a** IECs from *Gsdme*^−/−^ (KO) mice and wild-type (WT) littermates treated with DSS for 7 days were isolated; then, total RNA was isolated and the mRNA levels of DAMPs were determined by qPCR and normalized to GAPDH. **b**–**g** IECs from KO and WT littermates treated with DSS for 7 days were isolated, then washed with PBS containing Penicillin and Streptomycin for 3 times, and finally were plated in dishes with DMEM medium containing 10%FBS for 12 h. Cell medium was used for ELISA detection. **h**–**j** Isolated IECs from KO and WT littermates were given TNF-α (50 ng/ml) plus CHX (20 µg/ml) treatment for 12 h. **h**, **i** Culture supernatant was collected for ELISA and LDH release assays. **j** Representative immunoblot images of IECs. Data are shown as means ± SEM from six mice in each group. Data shown are representative of three independent experiments. *NS* not significant; **P < 0.01; ***P < 0.001

Due to the distinctive characteristic of membrane disruption, GSDME-mediated pyroptosis is believed to induce subsequent inflammatory responses through the release of intracellular DAMPs [14]. We investigated why GSDME may facilitate colitis development and proposed a role for DAMPs released from pyroptotic cells in promoting colitis development. Since GSDME was mainly expressed in the intestinal epithelium (Fig. 1d), we measured the intracellular expression levels of DAMPs in IECs and found higher levels of HMGB1 but not other DAMPs in IECs isolated from DSS-challenged WT mice than in IECs from *Gsdme*^−/−^ mice (Fig. 3a).

Extracellular HMGB1 can induce inflammatory responses [17]. We next determined the levels of extracellular HMGB1 released from IECs. After DSS exposure, we found higher levels of extracellular HMGB1 released by IECs from WT mice than IECs from *Gsdme*^−/−^ mice (Fig. 3b). In addition, the extracellular levels of other kinds of DAMPs were also determined by ELISA. The level of S100A9 but not S100A8, LTF, IL1a, or IL33 released by the IECs from DSS-challenged WT mice was significantly increased compared to that of IECs from *Gsdme*^−/−^ mice (Fig. 3c–g).

To further confirm the role of GSDME-mediated pyroptosis in HMGB1 release, we treated isolated IECs with TNF-α and CHX to induce pyroptosis [11]. In the absence of TNF-α and CHX, the levels of extracellular HMGB1 were low and were not significantly different between the WT and *Gsdme*^−/−^ groups. After treatment with TNF-α and CHX, the loss of GSDME significantly decreased HMGB1 release (Fig. 3h). In addition, treatment with TNF-α and CHX markedly increased LDH release and GSDME cleavage (Fig. 3i, j) and induced obvious pyroptotic bubbles (Additional file 1: Fig. S1), suggesting that the induction of pyroptosis by TNF-α and CHX was highly efficient. A previous study demonstrated that HMGB1 plays essential roles in the development of DSS-induced colitis [27]. Taken together, these results indicate that GSDME-mediated pyroptosis promotes mucosal inflammation through the release of HMGB1 by IECs.

### ***Gsdme***^***−/−***^*** mice are resistant to AOM/DSS-induced CAC***

**Fig. 4:**
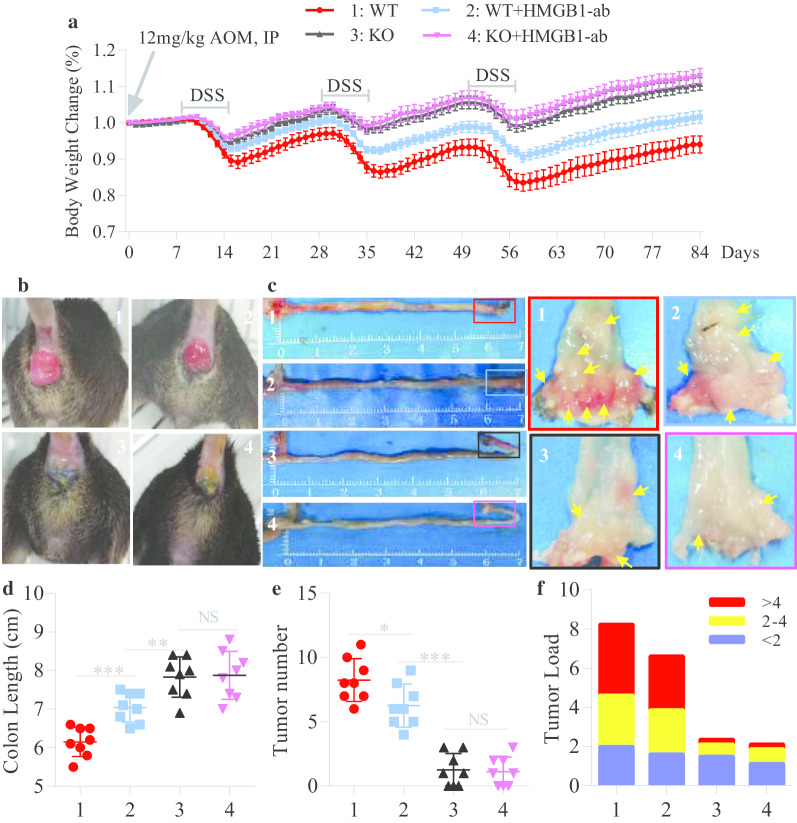
Inhibition of HMGB1 alleviates AOM/DSS-induced CAC. *Gsdme*^−/−^ (KO) mice and wild-type (WT) littermate controls were induced CAC with AOM/DSS as described above, and in this process, they were intraperitoneally injected with neutralizing anti-HMGB1 antibody (HMGB1-ab) at days 1, 3, 5 during DSS treatment (*n* = 8 per group). **a** Body weight change was measured daily. **b** Representative images of rectal prolapse at day 84 of the experimental procedure. **c**, **d** Representative images of the colon and the distal portion (arrows: tumor) and length values of the colon at day 84 of the experimental procedure. **e**, **f** The number of colonic tumors and the tumor size distribution according to tumor numbers (tumor load) were recorded. All data shown are representative of three independent experiments. *NS* not significant; *P < 0.05; **P < 0.01; ***P < 0.001

Patients that have IBD for a long time have a high risk for developing colitis-associated cancer (CAC) [28]. We subsequently investigated whether GSDME participates in the development of CAC. CAC was induced in *Gsdme*^−/−^ mice and WT littermate controls with AOM/DSS as previously described [19]. We observed that *Gsdme*^−/−^ mice exhibited reduced weight loss and colon shortening and attenuated rectal prolapse (Fig. 4a–d) compared to their WT littermates. Moreover, the numbers and sizes of macroscopically visible tumors from *Gsdme*^−/−^ mice were significantly decreased compared to those from their WT littermates (Fig. 4e, f). Importantly, histological examination showed a large number of adenocarcinomas in the mucosa and many malignant cells infiltrating the colonic submucosa of WT mice, but only some adenomas in the mucosa of *Gsdme*^−/−^ mice (Additional file 1: Fig. S2).

### HMGB1 contributes to CAC tumorigenesis

To explore the role of HMGB1 in CAC tumorigenesis, we first measured HMGB1 expression in mice with AOM/DSS-induced CAC. WT mice exposed to AOM/DSS had obvious GSDME cleavage and increased HMGB1 expression in IECs (Additional file 1: Fig. S3a–c). After GSDME was knocked out, HMGB1 expression was significantly decreased in the colon (Additional file 1: Fig. S3d–g). To further determine the function of HMGB1, we treated AOM/DSS-induced *Gsdme*^−/−^ mice and WT littermate controls with a neutralizing anti-HMGB1 antibody. We found obvious decreases in weight loss, colon shortening, rectal prolapse, and tumor numbers and sizes in antibody-treated WT mice compared to untreated WT mice (Fig. 4a–f). In addition, the neutralizing anti-HMGB1 antibody significantly decreased the protein expression of Ki67 and PCNA in the colons of AOM/DSS-challenged WT mice (Additional file 1: Fig. S4a–c). These results indicate that HMGB1 contributes to CAC tumorigenesis, which was consistent with a previous finding showing that a neutralizing anti-HMGB1 antibody dramatically decreased the tumor numbers in DSS-induced Apc^Min/+^ mice [27]. However, we also observed that antibody treatment did not significantly decrease the tumor numbers in *Gsdme*^−/−^ mice (Fig. 4e). This result suggests that in addition to HMGB1, there could be other tumor-promoting molecules released from cells through GSDME-mediated pyroptosis.

### HMGB1 induces tumor proliferation and PCNA expression through the ERK1/2 pathway

**Fig. 5:**
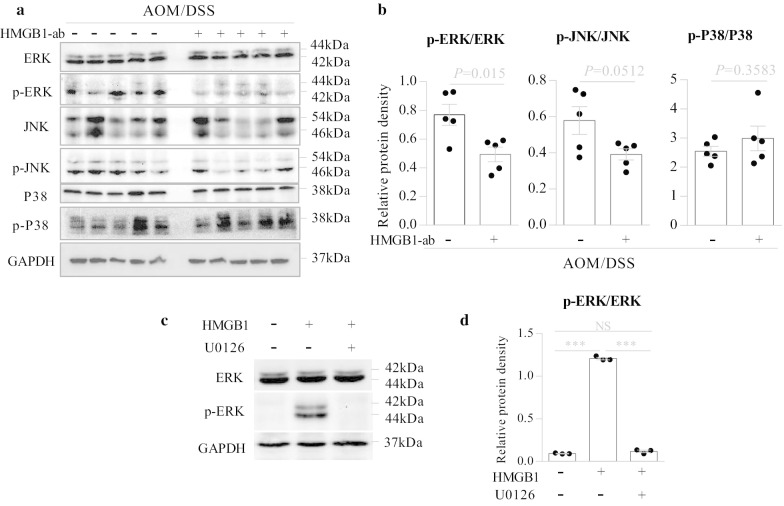
HMGB1 activates the ERK1/2 signaling pathway. **a**, **b** Wild-type (WT) mice were induced CAC with AOM/DSS as described above, and in this process, they were intraperitoneally injected with or without neutralizing anti-HMGB1 antibody (HMGB1-ab) at days 1, 3, 5 during DSS treatment. Tumor tissues in the colon were excised at day 84 of the experimental procedure, and whole tissue extracts from the tumors were prepared and analyzed for the phosphorylation of ERK1/2, JNK and P38 by western blotting. **a** The representative images. **b** Quantitative analyses of proteins. Data are shown as means ± SEM from five mice in each group. Data shown are representative of three independent experiments. **c**, **d** CT26 cells (1 × 10^5^ cells/well) were seeded in 6-well plates. After 24 h, the cells were incubated for 1 h with or without U0126 (10 μM) before stimulation with HMGB1 (1 μg/ml) for 12 h; then, whole cell extracts were analyzed for the phosphorylation of ERK1/2 by western blotting. **c** The representative images. **d** Quantitative analyses of proteins. Data are shown as means ± SEM from three independent experiments. *NS* not significant; ***P < 0.001

**Fig. 6:**
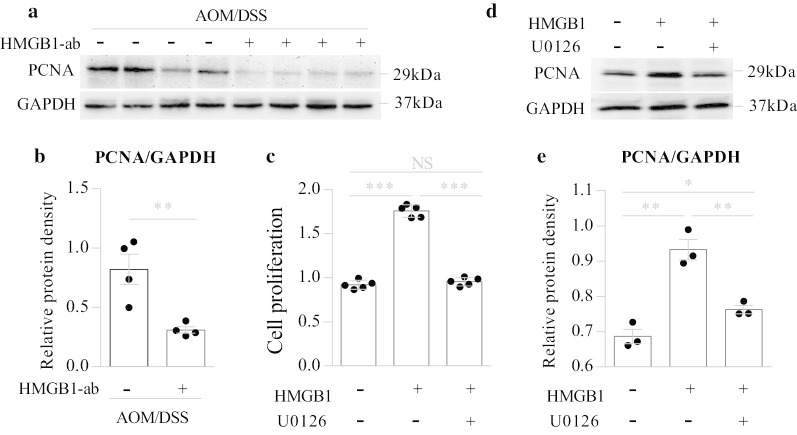
HMGB1 induces tumor proliferation and PCNA expression through the ERK1/2 pathway. **a**, **b** Wild-type (WT) mice were induced CAC with AOM/DSS as described above, and in this process, they were intraperitoneally injected with or without neutralizing anti-HMGB1 antibody (HMGB1-ab) at days 1, 3, 5 during DSS treatment. Tumor tissues in the colon were excised at day 84 of the experimental procedure, and whole tissue extracts from the tumors were prepared and analyzed for PCNA expression by western blotting. **a** The representative images. **b** Quantitative analyses of proteins. Data are shown as means ± SEM from four mice in each group. Data shown are representative of three independent experiments. **c** CT26 cells (1 × 10^3^ cells/well) were seeded in 96-well plates. After 24 h, the cells were incubated for 1 h with or without U0126 (10 μM) before stimulation with HMGB1 (1 μg/ml) for 48 h, and then, the cell proliferation activity was measured by CCK-8 assay. Data are shown as means ± SD from five wells in each group. Data shown are representative of three independent experiments. **d**, **e** CT26 cells (1 × 10^5^ cells/well) were seeded in 6-well plates. After 24 h, the cells were incubated for 1 h with or without U0126 (10 μM) before stimulation with HMGB1 (1 μg/ml) for 48 h, and then, whole cell extracts were analyzed for PCNA expression by western blotting. **d** The representative images. **e** Quantitative analyses of proteins. Data are shown as means ± SEM from three independent experiments. *NS* not significant; *P < 0.05; **P < 0.01; ***P < 0.001

A previous study demonstrated that blocking the RAGE-HMGB1 axis suppresses the growth and metastases of C6 glioma cells by inhibiting the activation of mitogen-activated protein (MAP) kinases, including ERK1/2, p38 and JNK [29]. Hyperactivation of the ERK1/2 pathway, which is composed of the kinases RAS, RAF, MEK, and ERK1/2, drives cancer cell growth in a wide array of cancers [30, 31]. We therefore assessed whether the MAPK pathway contributed to HMGB1-mediated CAC tumorigenesis. We found that the neutralizing anti-HMGB1 antibody significantly decreased ERK1/2 activation (Fig. 5a, b) and PCNA expression (Fig. 6a, b) in AOM/DSS-induced WT mice.

To further investigate the effect of the ERK1/2 pathway on HMGB1-induced cancer cell proliferation, the ERK1/2 inhibitor U0126 was used. In the in vitro experiments, CT26 cells were incubated for 1 h with U0126 before stimulation with HMGB1 for 48 h, and then, cell proliferation was measured by CCK-8 assays. We found that U0126 completely suppressed HMGB1-induced ERK1/2 activation (Fig. 5c, d), cell proliferation and PCNA expression (Fig. 6c–e). Similar results were observed in vivo (Additional file 1: Fig. S5a–e). These results suggest that HMGB1 may participate in CAC tumorigenesis through the ERK1/2 pathway.

## Discussion

The most serious complication of IBD is CAC, and one of the hallmarks of CAC is chronic inflammation [7]. Although inflammation has been identified as a tumor-promoting mechanism in CAC induction [7], the precise details of this mechanism are still unclear. This study was focused on the precise mechanism by which GSDME-mediated pyroptosis participates in the development of experimentally induced CAC in mice. Our results show that GSDME-mediated pyroptosis and the subsequent release of HMGB1 are associated with CAC tumorigenesis. Mechanistically, our results show that HMGB1 induces CAC tumorigenesis and PCNA expression through the ERK1/2 signaling pathway. PCNA is a chief proliferation marker that reflects the level of cell proliferation [32, 33]. Our finding is consistent with a previous report showing that blocking the RAGE-HMGB1 axis suppresses the growth and metastases of C6 glioma cells by inhibiting activation of the MAPK pathway [29].

DAMPs, which are damaged tissue-derived proinflammatory mediators such as HMGB1, S100 proteins, and IL1α, may trigger chronic inflammation and thus promote the development of chronic inflammation-related tumors [34]. Recent studies have revealed that the plasma membranes of pyroptotic cells rupture and release DAMPs [16, 35]. In this study, we found that genetic deletion of GSDME, an important executor protein of pyroptosis, could effectively decrease HMGB1 expression and release from colonic tissues in a DSS-induced colitis model. Furthermore, by using anti-HMGB1 therapy in the CAC model, we further determined the role of HMGB1 in promoting CAC tumorigenesis. Our findings suggest that HMGB1 might serve as a novel and attractive therapeutic target for future clinical treatment of CAC.

Interestingly, our data showed that GSDME-deficient mice had a better prognosis than WT mice treated with the neutralizing anti-HMGB1 antibody but had a nonsignificant difference from GSDME-deficient mice treated with neutralizing anti-HMGB1 antibody. These results suggest that GSDME is more important than HMGB1 in CAC tumorigenesis. The limited effect of anti-HMGB1 in this study may have been due to an insufficient dose or frequency. Another possibility is that other DAMPs released from GSDME-mediated pyroptotic cells might also contribute to CAC tumorigenesis.

Notably, a recent study using an AOM-induced CRC model showed that there were no major differences between *Gsdme*^−/−^ and WT mice regarding the number of mice bearing microscopic proliferative lesions nor the number of proliferative lesions per mouse [36]. However, there was a trend toward more affected mice and proliferative lesions in WT mice than in *Gsdme*^−/−^ mice [36]. To investigate why this finding was different from ours, we hypothesize that the discrepancy could be due to the use of different chemically induced colorectal cancer models and different WT controls. In our study, we used an AOM/DSS-induced CAC model and WT littermate controls, while the other group used an AOM-induced CRC model and WT nonlittermate controls. However, whether this is indeed the case remains to be further studied.

## Conclusions

In summary, we show that GSDME-mediated pyroptosis contributes to the development of CAC by releasing intracellular HMGB1, which induces tumor cell proliferation and PCNA expression through the ERK1/2 pathway. Our findings highlight the emerging role of GSDME-mediated HMGB1 secretion in promoting CAC tumorigenesis and provide new insights for the future development of CAC therapeutic strategies by inhibiting GSDME-mediated pyroptosis or using a neutralizing anti-HMGB1 antibody. Future studies are needed to investigate (1) the inflammatory immune response triggered by HMGB1 and its effect on the CAC environment; (2) other DAMPs besides HMGB1 that contribute to CAC development; and (3) the implications for the involvement of other forms of cell death, such as necroptosis and ferroptosis, in CAC tumorigenesis.

## Supplementary information

**Additional file 1:** Supplementary figures and tables.

## Data Availability

The data sets supporting the conclusions of this article are included within the article.

## Abbreviations

AOM: Azoxymethane
CAC: Colitis-associated colorectal cancer
CD: Crohn’s disease
DSS: Dextran sodium sulfate
GSDME: Gasdermin E
HMGB1: High mobility group box protein 1
IBD: Inflammatory bowel disease
IEC: Intestinal epithelial cell
IHC: Immunohistochemical
PCNA: Proliferating cell nuclear antigen
PCR: Polymerase chain reaction
TNF-α: Tumor necrosis factor α
UC: Ulcerative colitis
