# Supplementary material for: HMGB1 released from GSDME-mediated pyroptotic epithelial cells participates in the tumorigenesis of colitis-associated colorectal cancer through the ERK1/2 pathway
Source: J Hematol Oncol. 2020 Nov 7;13:149. doi: 10.1186/s13045-020-00985-0 (PMC7648939; doi:10.1186/s13045-020-00985-0)
Supplement: Supplementary file 1 — Additional file 1: Supplementary figures and tables. [file 13045_2020_985_MOESM1_ESM.docx]

**HMGB1 released from GSDME-mediated pyroptotic epithelial cells participates in the tumorigenesis of** **colitis-associated colorectal cancer through the ERK1/2 pathway**

**Additional file 1: Figures and tables**


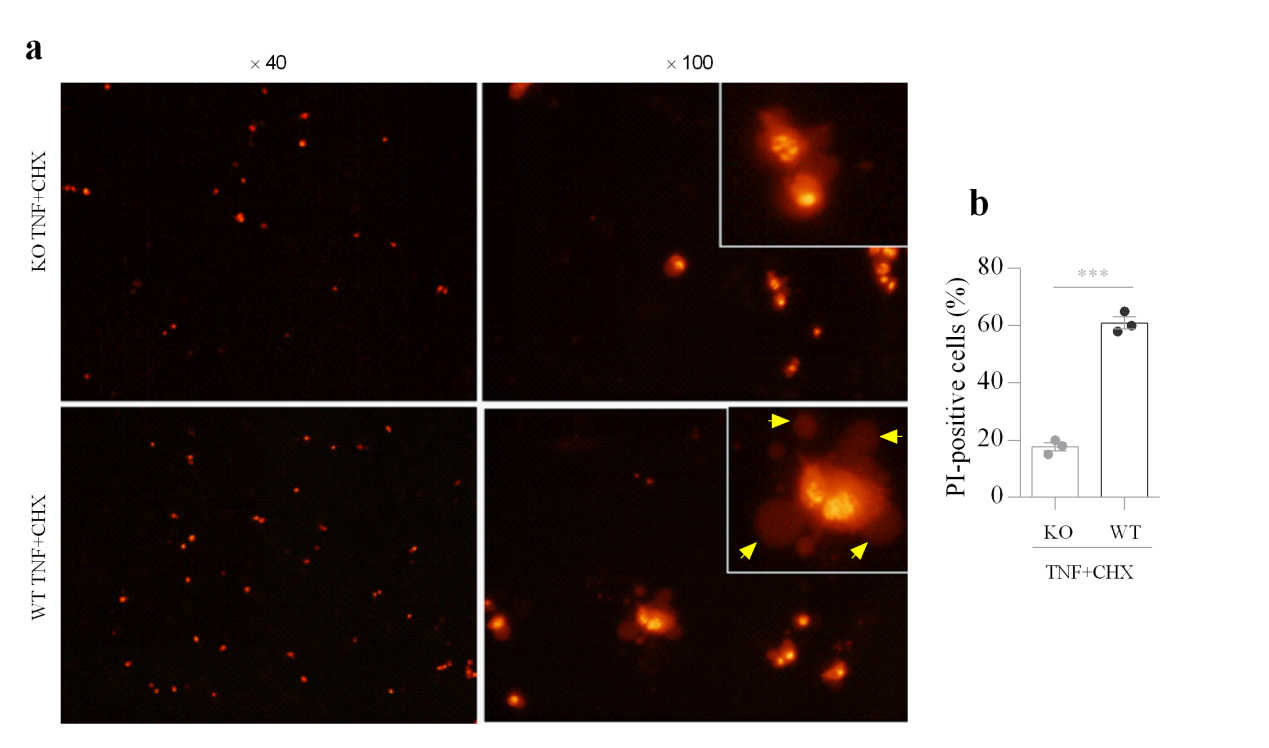


**Additional file 1: Figure S1 GSDME determines pyroptosis in IECs in response to** **TNF-α plus CHX**

Isolated IECs from *Gsdme*^-/-^ (KO) and WT littermates were given TNF-α (50 ng/ml) plus CHX (20 ug/ml) treatment for 12 hours. Then , the cells were stained with [propidium iodide](https://www.so.com/link?m=bXroN7vXkoiLt7b2rI5PstR15czCCxT4f62Asj5shOBQWuJpmxYu3QF0VoAdEfSa85euvIjGpoRVBee11pFE3n1RH%2BI90AsO%2FoX8Ndur8wqF11w1u2GWFlATbz0b28grPhWjpPu3NbNXLS29Oa%2BxnNBWNvM1wvMcBHpOo%2FMKfZ%2FRTF51eb8SQ18j7BuJmcS%2FP0fXoIHYfnIo%3D) (PI) solution (2 ug/ml PI) and immediately observed under the Olympus microscope. (**a**) Representative images of PI-stained cells. Yellow arrows: pyroptotic bubbles. Original magnification: ×40, ×100. (**b**) PI-positive cells are expressed as mean ± SD from three technical replicates. All data shown are representative of three independent experiments. ****P*<0.001.

**
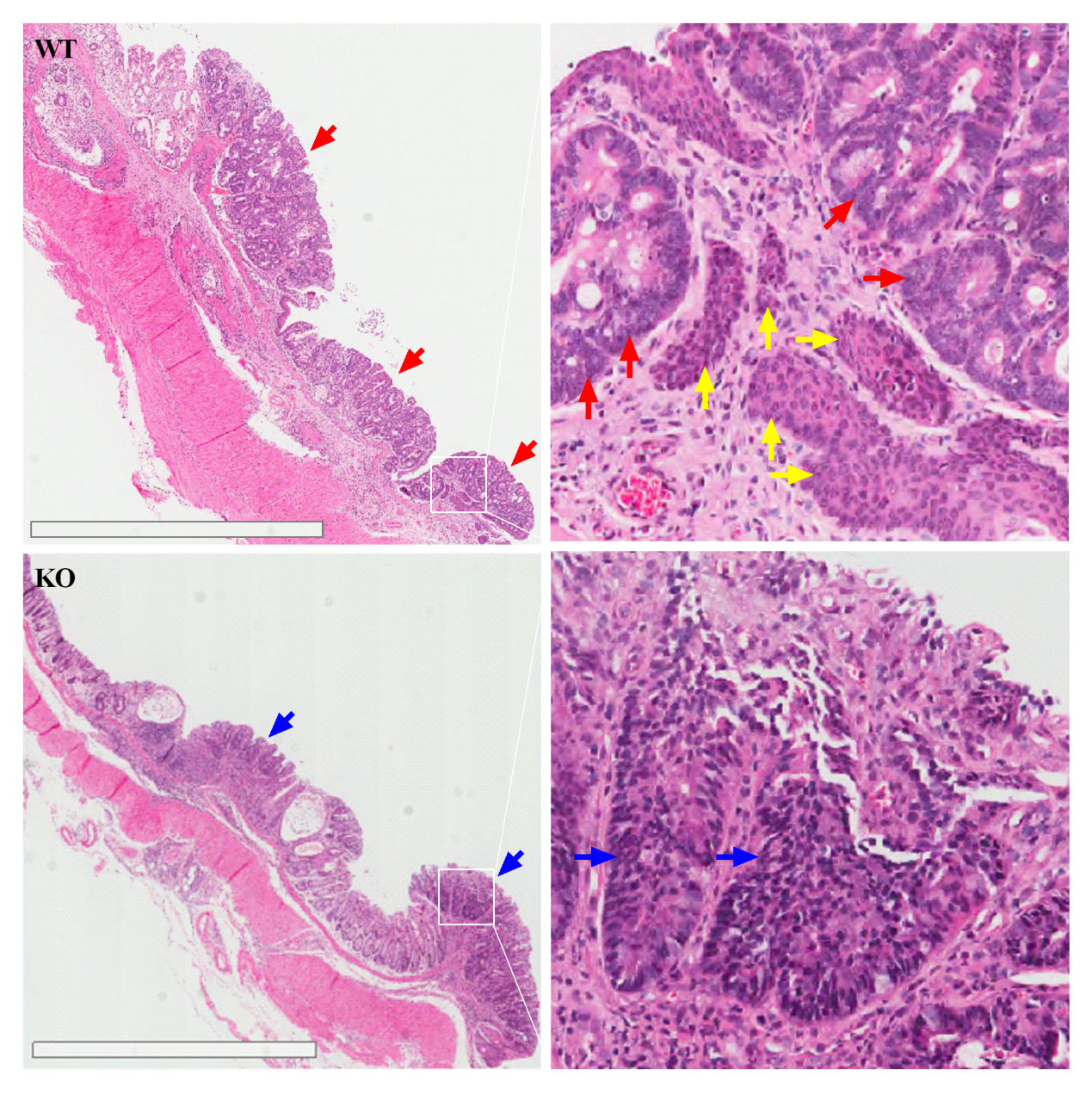
**

**Additional file 1: Figure S2 *Gsdme*^-/-^ mice are resistant to AOM/DSS-induced CAC**

*Gsdme*^-/-^ (KO) mice and Wild-type (WT) littermate controls were induced CAC with AOM/DSS as described above. Representative HE-stained sections of the colon collected at day 84 of the experimental procedure. Scale bars: 2mm. Red arrows: adenocarcinomas. Yellow arrows: malignant cells infiltrating into the submucosa. Blue arrows: adenomas.


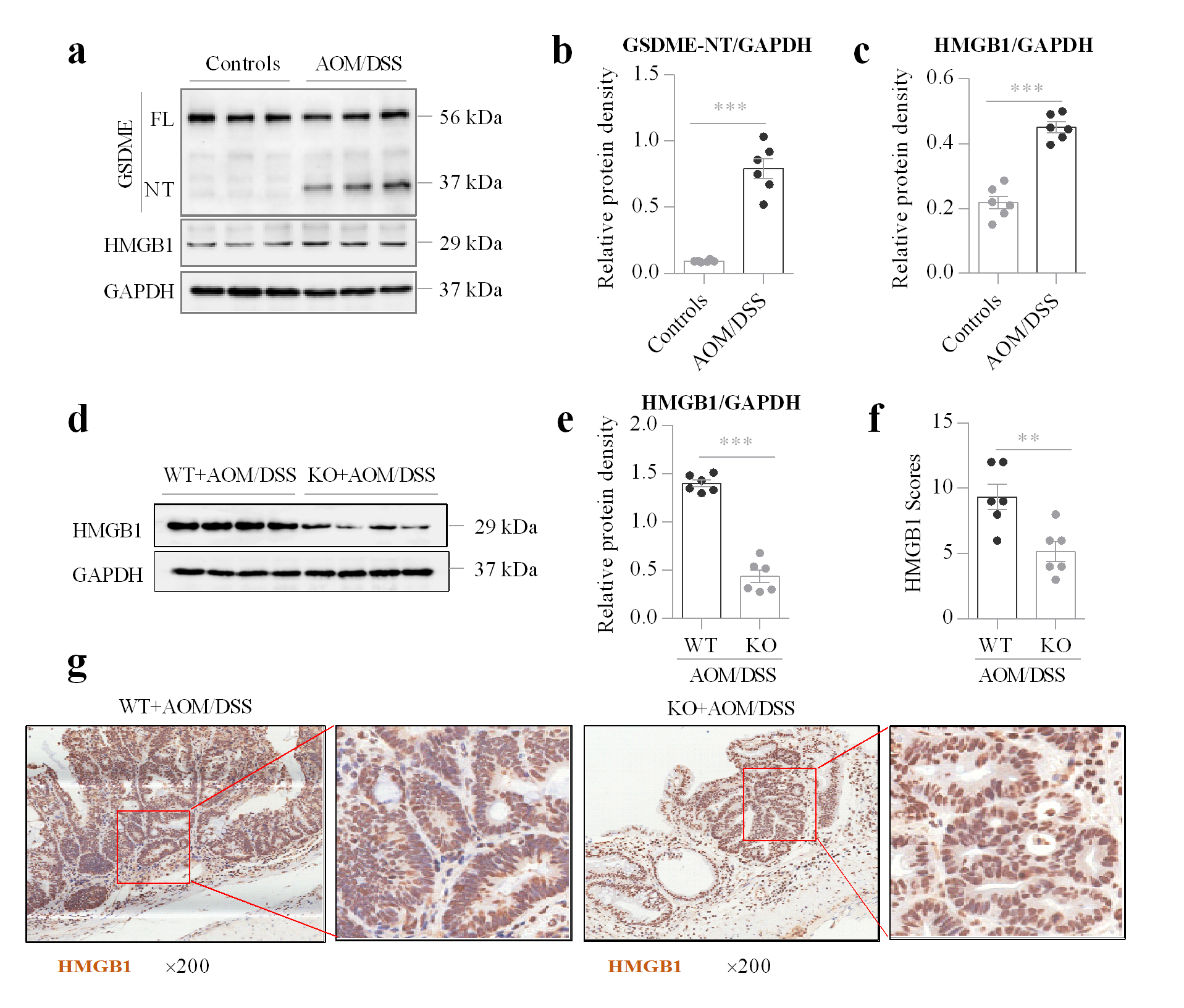


**Additional file 1: Figure S3 HMGB1 expression is increased in CAC**

*Gsdme*^-/-^ (KO) mice and Wild-type (WT) littermate controls were induced CAC with AOM/DSS as described above. (**a-c**) Immunoblot analyses of GSDME cleavage and HMGB1 expression in the IECs. (**a**) Representative immunoblot images. The full length of GSDME (GSDME-FL) is shown in 56 kDa and GSDME-NT in 37 kDa. (**b, c**) Quantitative analyses of proteins. (**d, e**) Immunoblot analyses of HMGB1 expression in the colons. (**d**) Representative immunoblot images. (**e**) Quantitative analyses of HMGB1. (**f, g**) IHC analyses of HMGB1 expression in the colons. (**f**) Quantitative analyses of HMGB1. (**g**) Representative IHC images. Original magnification: ×200. Data are shown as means ± SEM from six mice in each group. Data shown are representative of three independent experiments. ***P*<0.01, ****P*<0.001.


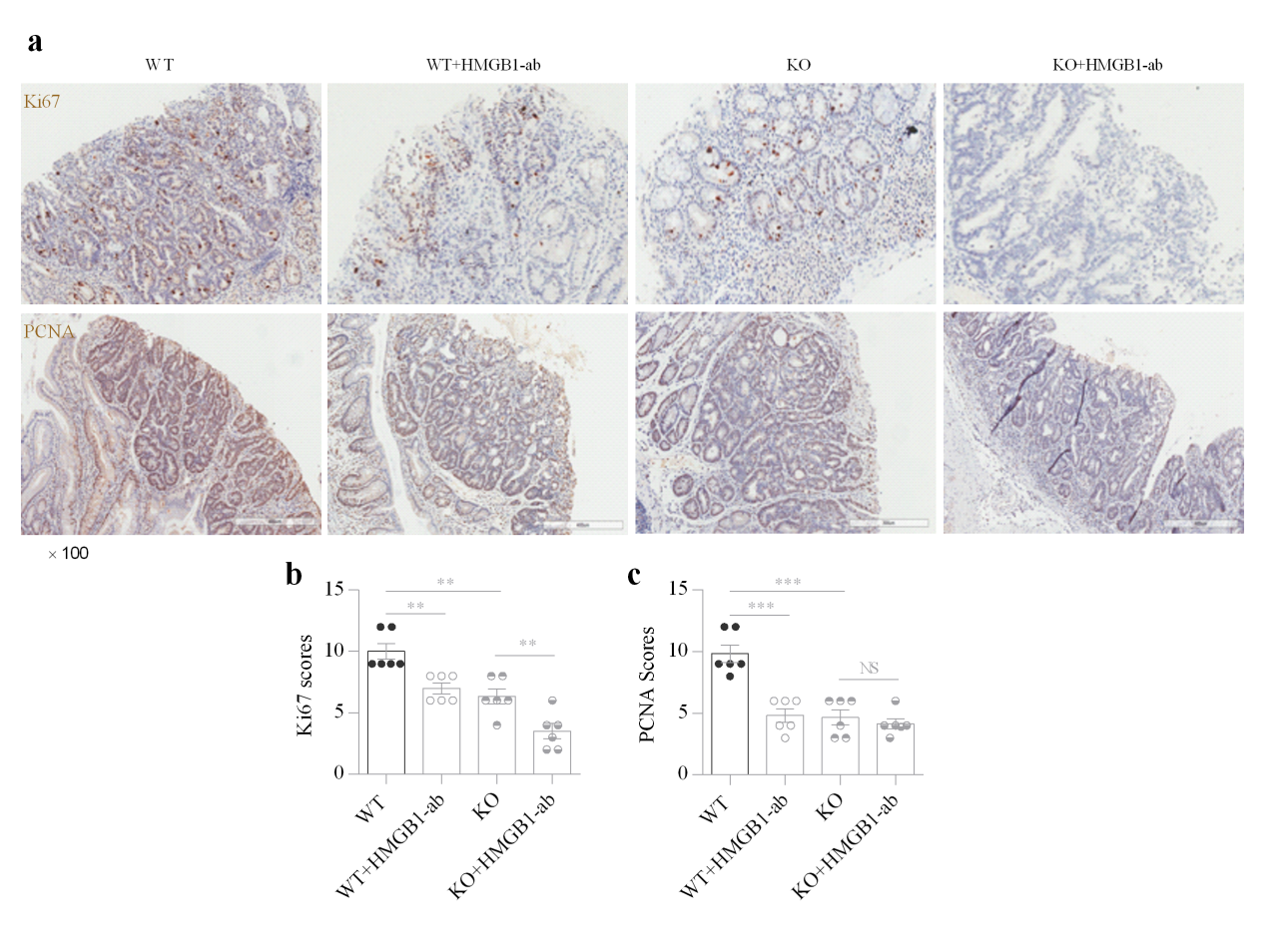


**Additional file 1: Figure S4 HMGB1 in****creases Ki67 and PCNA expression in CAC**

*Gsdme*^-/-^ (KO) mice and Wild-type (WT) littermate controls were induced CAC with AOM/DSS as described above, and in this process, they were intraperitoneally injected with neutralizing anti-HMGB1 antibody (HMGB1-ab) at days 1, 3, 5 during DSS treatment. IHC analyses of Ki67 and PCNA expression in the colons. (**a**) Representative IHC images. Original magnification: ×100. (**b, c**) Quantitative analyses of Ki67 and PCNA expression. Data are shown as means ± SEM from six mice in each group. Data shown are representative of three independent experiments. NS, not significant; ***P*<0.01, ****P*<0.001.


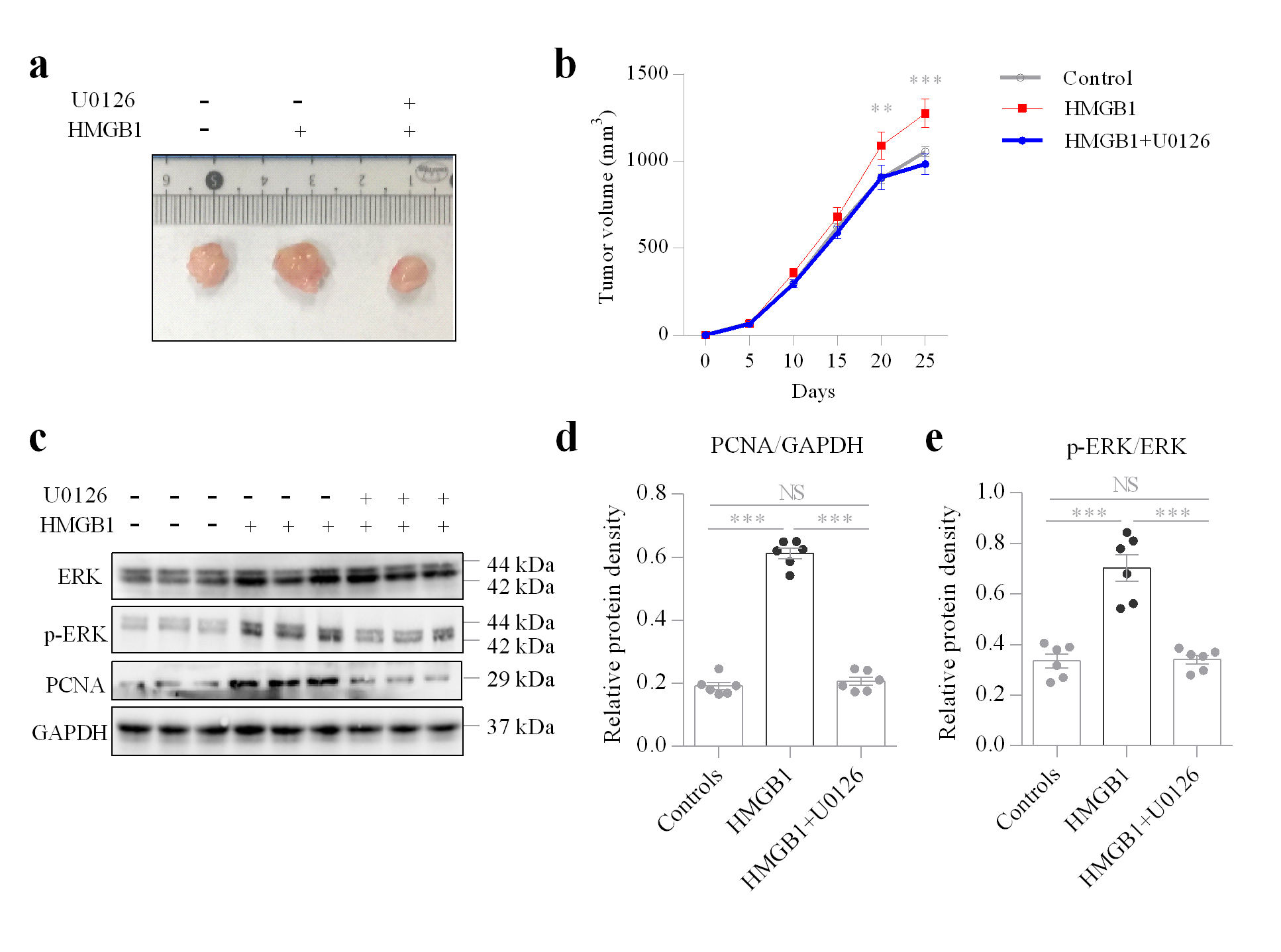


**Additional file 1: Figure S5 U0126 suppresses HMGB1-induced cell proliferation, ERK1/2 activation and PCNA expression *in vivo***

(**a-e**) Eight-week-old male Balb/c mice were randomly divided into Control group and HMGB1 group and HMGB1+U0126 group (n=6). 1×10^7^ CT26 cell were suspended in 100ul PBS and injected into the hind leg of mice. On day 0, 5, 10, 15, 20, mice were injected intraperitoneally with sterile saline (Control), recombinant mouse HMGB1 protein (1ug/ml) and HMGB1 plus U0126 (15 mg/kg). 3 mg/ml U0126 solution containing 5% DMSO, 40% PEG-300, 5% Tween 80 and 50% sterile saline was used in this study. (**a, b**) Mice were sacrificed on day 25 and tumor volume were measured per 5 days during the process. (**c-e**) ERK1/2 activation and PCNA expression were determined by western blot. (**c**) Representative western blot images. (**d, e**) Quantitative analyses of p-ERK and PCNA expression. Data are shown as means ± SEM from six mice in each group. Data shown are representative of three independent experiments. NS, not significant; ****P*<0.001.

**Additional file 1: Table S1 Demographic characteristics of the study population**

|  | **IBD** | **Controls** |
| --- | --- | --- |
| **Number** | 85 | 40 |
| **Male, n (%)** | 40 (47.05%) | 20 (50.0%) |
| **Age (years)** | 42.15±15.65 (15-70) | 35.25±12.85 (16-65) |
| **Disease duration (months)** | 38.8±52.6 (0-248) | _ |
| **Age at diagnosis (year)** | 42.18±15.02 (16-68) | _ |

**Additional file 1: Table S2 Primer sequences for qPCR**

| **Gene** | **Fw_sequence** | **Rv_sequence** |
| --- | --- | --- |
| IL1α | GCACCTTACACCTACCAGAGT | AAACTTCTGCCTGACGAGCTT |
| IL33 | TGAGACTCCGTTCTGGCCTC | CTCTTCATGCTTGGTACCCGAT |
| HMGB1 | GGCGAGCATCCTGGCTTATC | GGCTGCTTGTCATCTGCTG |
| S100A8 | GACAATGCCGTCTGAACTGG | GCTACTCCTTGTGGCTGTCTT |
| S100A9 | ACCACCATCATCGACACCTTC | AAAGGTTGCCAACTGTGCTTC |
| LTF | ATTTCCTGTGATAACCCACTGTG | GTGGACTTTACCTTTGAGCTGG |
| GAPDH | TGTGTCCGTCGTGGATCTGA | TTGCTGTTGAAGTCGCAGGAG |
